# Laparoscopic Cholecystectomy: Evaluation of Web-Based Information

**DOI:** 10.7759/cureus.20897

**Published:** 2022-01-03

**Authors:** Sreelakshmi Mallappa, Ramawad Soobrah

**Affiliations:** 1 Department of General and Colorectal Surgery, The Hillingdon Hospitals NHS Foundation Trust, Uxbridge, GBR; 2 Department of Breast Surgery, Wellkin Hospital, Moka, MUS

**Keywords:** information quality, healthcare information, internet, readability, laparoscopic cholecystectomy

## Abstract

Introduction

Laparoscopic cholecystectomy (LC), the gold standard treatment for symptomatic gallstone disease, is the most common procedure performed by general surgeons worldwide. The internet remains to be a popular source of medical information. Our aim was to evaluate the quality and readability of information available on the web for patients undergoing LC and to compare the information provided by the National Health Service (NHS) and non-NHS websites.

Methods

We searched for the keywords ‘laparoscopic cholecystectomy’ using the three most popular search engines (Google, Yahoo and MSN) and looked at the first 50 websites only. The readability of each document was assessed using the Flesch Reading Ease (FRE) score. We checked Health on the Net Foundation Code of Conduct (HONcode) certification status, whether the sites had been checked by an expert and when the information was last updated.

Results

Fifty-five of the possible 150 sites were analysed thus excluding repetitions (n=65), irrelevant content (n=26) or inaccessible links (n=3). Only seven of those were HONcode-certified. The mean FRE score was 46 (range 0-68, SD=16.13). There were 13 NHS sites and 42 non-NHS sites. The mean FRE score for the NHS sites was significantly better compared to the non-NHS sites [58.31 (SD=5.01) vs 42.21 (SD=16.35); p=0.001]. Fifty-four per cent (54%) of the analysed websites had been checked by a medical expert and 22% were updated within the last year.

Conclusions

This study highlights the poor quality and readability of information on medical websites. The information provided by NHS sites have significantly better readability compared to non-NHS sites.

## Introduction

The Internet has become an important tool for many people with health concerns [1-2]. A vast majority of Internet users find it easy to search for online medical information. Such information is not always accurate or easily comprehensible. The accuracy and completeness of materials available on the Internet have been a matter of concern [3-5]. A report published by the U.S. Federal Trade Commission in 2001 identified hundreds of websites making questionable, deceptive health claims for products sold to treat serious diseases [6]. Approximately 8% of the adult population, or more than 5.5 million people in the United Kingdom, have gallstones [7]. About 50,000 of these patients undergo gallbladder surgery each year. A majority of patients with gallstones remain asymptomatic, but a minority may develop complications, ranging from biliary colic to life-threatening complications such as acute infective complications or pancreatitis [7]. Laparoscopic cholecystectomy is the most common procedure performed by general surgeons worldwide. Our aim was to evaluate the quality and readability of information available on the web for patients undergoing laparoscopic cholecystectomy.

## Materials and methods

We searched for the keywords ‘laparoscopic cholecystectomy’ using Google™, Yahoo!™ and MSN/Bing™, the three most commonly used search engines (Table 1) [8]. Both authors identified and reviewed the sites in a single search session. The majority of Internet users only browse through the first 10 sites listed by search engines; in fact, 99% do not search beyond the first 50 websites, and only a small percentage of patients use search modifiers [9-10]. Hence, no restrictions were applied to our search strategy and we looked at the first 50 websites only for each search engine. After analysing the contents of all the selected sites, we assigned each to one of the following categories: "commercial", "healthcare professionals," or "patients" (Table 2). We also checked when the information provided on the site was last updated and whether the information provided by the sites was referenced.

**Table 1:** Top five most popular search engines, March 2021

Provider	Estimated unique monthly visitors
Google	1, 800, 000, 000
Bing	500, 000, 000
Yahoo! Search	490, 000, 000
Baidu	480, 000, 000
Ask	300, 000, 000

**Table 2:** Website categories

Category	Number of sites
Commercial	16
Healthcare professionals	4
Patients	35

Flesch Reading Ease (FRE) Score

Readability can be defined as ‘the ease of understanding or comprehension due to the style of writing’ [11]. There are currently over 200 readability formulas, and although not 100% accurate, they give a rough estimate of the reading skill required to read a text [12]. Readability formulas have been in use since the 1920s to predict the difficulty level of a text [13]. The Flesch’s Reading Ease (FRE) formula is one of the most tested and reliable readability formulas [10]. The FRE score varies from 0 to 100 [13], with low scores indicating complex documents while scores between 61-70 represent a standard readability level (Table 3) [11]. We used Microsoft Word® (Microsoft Corporation, Washington, DC) to calculate the FRE scores of the relevant websites.

**Table 3:** Flesch Reading Ease score interpretation

FRE Score	Reading Difficulty	Type of Magazine
91 – 100	Very easy	Comics
81 – 90	Easy	Pulp fiction
71 – 80	Fairly Easy	Slick fiction
61 – 70	Standard	Digests
51 – 60	Fairly difficult	Quality
31 – 50	Difficult	Academic
0 – 30	Very difficult	Scientific

Health on the Net Foundation Code of Conduct (HONcode)

**Figure 1:**
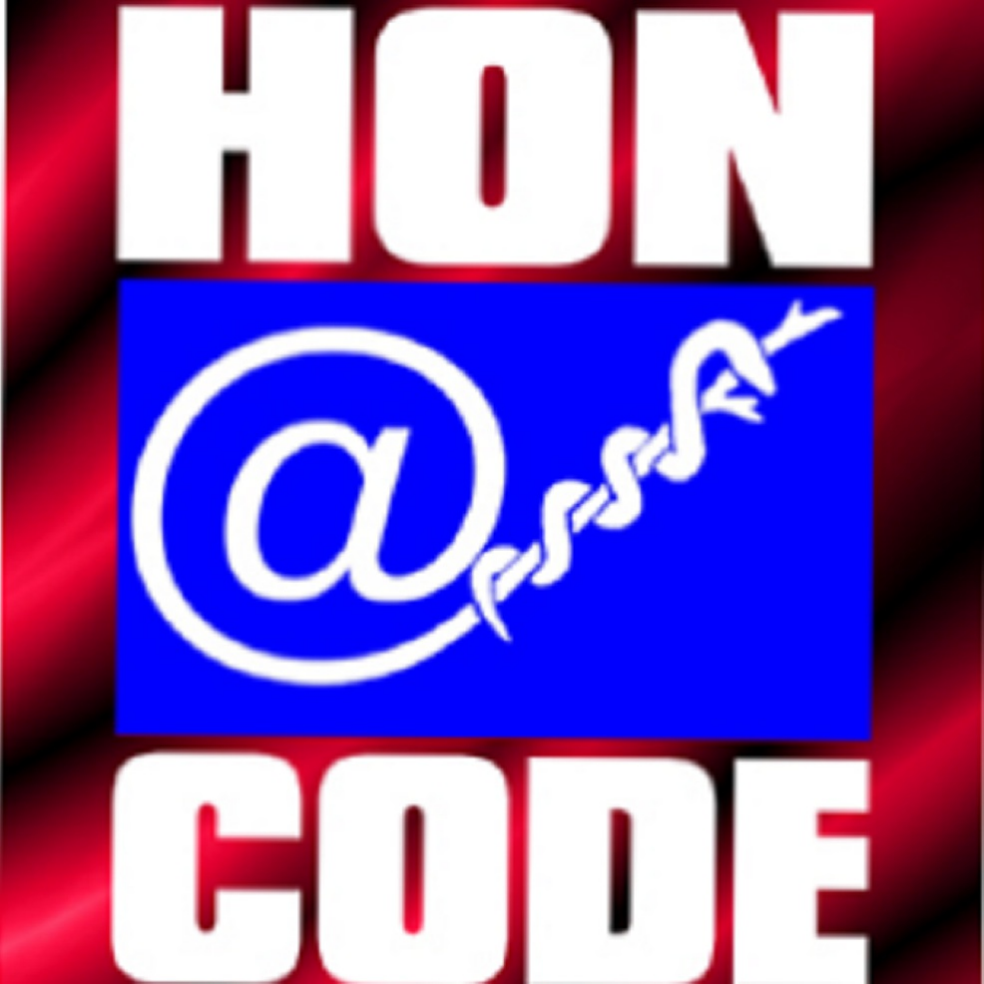
HONCode logo Health on the Net Foundation Code of Conduct: HONcode

Medical and health information available on the Internet should be reliable. The Health On the Net (HON) Foundation is an international organisation that aims to standardize the health information available on the Web to make it a reliable source for its users [14]. The HON Foundation has set out eight HONcode principles (Table 4). Subscribing sites will have to adhere to the HONcode principles to display the HONcode seal (Figure 1).

**Table 4:** HONcode principles Health on the Net Foundation Code of Conduct: HONcode

1. Authority: Give qualifications of authors	5. Justifiability: Justification of claims/balanced and objective claims
2. Complementarity: Information to support, not replace	6. Transparency: Accessibility, provide valid contact details
3. Confidentiality: Respect the privacy of site users	7. Financial disclosure: Provide details of funding
4. Attribution: Cite the sources and dates of medical information	8. Advertising: Clearly distinguish advertising from editorial content

Statistical analysis

We used Microsoft Office Excel® (Microsoft Corporation, Redmond, WA) to analyse the data gathered from the three search engines. An independent samples t-test was used to compare the means, and statistical significance was set at a *p-*value of <0.05.

## Results

**Figure 2:**
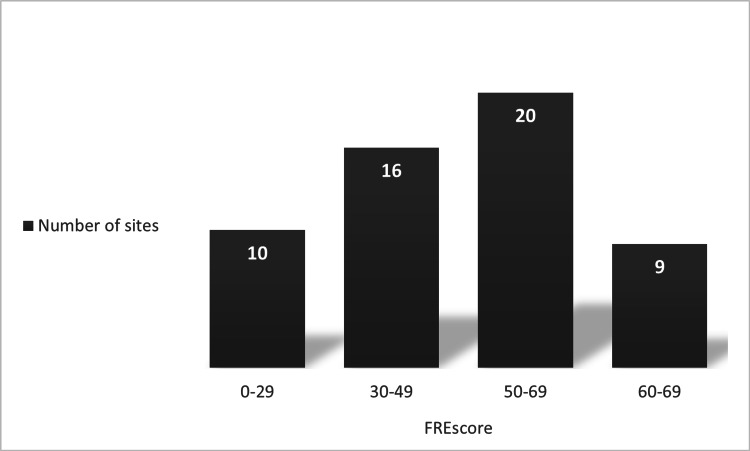
FRE score showing only nine of the analysed sites had standard readability levels Flesch Reading Ease: FRE

Of the 150 possible sites, only 55 were analysed. Others were excluded because of repetitions (n=65), irrelevant content (n=27) or inaccessible links (n=3). The mean FRE score for all the analysed sites was 46 (range 0 - 68, SD = 16.13) and only nine of those had a standard readability level (Figure 2). Thirty sites (64%) were classified as ‘difficult’ or ‘fairly difficult’ to read. Fifty-four per cent (54%) of the analysed websites had been checked by a medical expert and 22% had been updated within the last year. Nearly half (49%) of the websites analysed provided no data on when the information supplied was last updated. Table 2 illustrates the number of sites assigned to each category. Websites classified as ‘academic’ were excluded from the final analysis because it was felt that patients are unlikely to scrutinize those sites to obtain information about laparoscopic cholecystectomy. Twenty-seven sites were not analysed because we deemed the information provided by these sites to be irrelevant to patients.

Seven of the 55 websites analysed were HONcode-certified. There was no statistically significant difference between the FRE scores of HONcode-certified and HONcode-uncertified websites (44.3 v 46.3, p = 0.74). All of those HONcode-certified sites had been checked by a medical expert; however, none of them belonged to an NHS organisation. Five sites were designed for patient use, one for healthcare professionals and one was commercial in nature (private clinic) (Table 2). Four sites had been updated within the last year, two had been updated over two years ago and there was no mention of an update on one site. Only four of those sites provided relevant references.

Thirteen of the analysed websites were designed for NHS Trust Hospitals. The mean FRE score for the NHS sites was significantly higher compared to non-NHS sites [58.3 (SD=5.01) vs 42.2 (SD=16.3); p=0.001], indicating that the information provided by the NHS sites was less complex to understand compared to information provided by non-NHS sites. However, only three of those were of “standard” readability level. Moreover, out of the 13 sites, only three specified whether a medical expert had checked the information supplied and none were properly referenced. Two sites were updated within the last year, six over two years and four sites had no information when they were updated.

## Discussion

The Internet is an important source of health information for the layperson [15-17]. People have used the Internet for many years to access health-related information. It is estimated that eight in 10 internet users go online for health information [18]. A 37% increase over two years was observed in the Harris Interactive study, which estimated 160 million adults in the United States of America searched for health information online, which was an increase from 136 million in 2006 and 117 million in 2005 [19]. As the audience for accessing online medical information grows rapidly, so does the concern about the quality of medical information made available on the Internet [5]. The quality of information available on the Internet is variable, and studies have shown that the information on the Internet is often incomplete and sometimes dangerous [5,15,20-21]. Health-related websites are now amongst the most frequently accessed sites on the Internet with current estimates indicating that there are now over 100,000 sites offering health-related information [22]. A majority of health information seekers do not regularly check the quality indicators of the information they find online [18].

The information provided by a site should be comprehensive, relevant, and unbiased [23]. Currency and content production are some of the criteria used to assess reliability. A site will also need to provide a statement on how the information was produced and the quality check procedures undertaken. A site will have to update its content regularly with recommended six-monthly updates for treatment and longer updates for diagnosis and background information [24].

Quality criteria: simple codes of conduct

The e-Health Code of Ethics adopted in 2000 by the Internet Health Coalition is a well-known ‘code of conduct;’ the purpose of which is to offer a process of self-assessment by health site providers [25]. A code of conduct is a self-applied quality label where an organisation that has developed the code allows for those who abide by the terms of the code to display a label, seal or logo certifying compliance with the code [25]. The oldest, and perhaps best known, of such labels is the Health on the Net Foundation (HON) label whose eight-point set of quality criteria is currently used by more than 7000 Internet sites worldwide [14].

The Health on the Net Foundation critically reviews the contents of medical websites to improve the quality of healthcare information available on the Internet. Sites that comply with the benchmarks proposed by the HON Foundation can display the HONcode seal. At the end of the certificate validity, HONcode reviewers undertake a re-evaluation process to verify that these sites still respect the HONcode principles. Full conformity with all eight principles is indispensable for the prolongation of the certificate. The HONcode toolbar can also be downloaded and added to a web browser. It connects in real-time to the HON server and automatically checks the certification of the website being viewed. The HONcode seal appears in colour if the website is fully compliant. Hence, this seal on subscribing sites helps users identify sources of reliable information (Figure 1). Moreover, the certification is valid for a year, after which a re-evaluation of the website has to be undertaken. To date, HONcode is used by more than 7000 certified websites in over 100 countries [26].

Limitations

Our study has some limitations. FRE scores can be calculated using various online instruments and computer packages and the scores will vary depending on the software used. We chose Microsoft Word® because it is well-known software. What we are presenting is a snapshot of the problem. Website rankings change on a daily basis and the results may vary if the study were to be undertaken at a different time point. Factors affecting comprehension such as writing style and explanation of medical jargon are not accounted for when calculating the readability scores [27]. Eighty-three per cent of the analysed websites in our study were not accredited by HON Foundation. This does not necessarily imply that these sites do not adhere to the HON principles. It is possible that those sites may not have formally submitted an application for certification [28]. 

Suggestions for improvement

With the ever-growing reliance of the general public on gaining online healthcare information, urgent measures need to be taken to ensure high-quality information is made available. Healthcare providers should direct patients to known reputable sites with materials written at a suitable reading level. Healthcare organisations should provide disease-specific information leaflets with links to websites that provide useful patient-orientated information. High-quality medical websites can improve their visibility by using measures such as Search Engine Optimisation (SEO) [29].

## Conclusions

The Internet has been a readily available source for accessing health-related information for many years. However, little has been done to assess, control and assure the quality of this medical information. It is not easy for patients to judge the accuracy and credibility of information available, and the majority of health information is not easily comprehensible. This study has shown that the search for easily comprehensible, high-quality, reliable information about laparoscopic cholecystectomy on the Internet is challenging. There is an urgent need to develop clear, easily accessible, and authoritative resources for patients. Healthcare professionals have a duty to help patients identify such resources. It is reassuring to know that the information about laparoscopic cholecystectomy provided by NHS sites has better readability compared to non-NHS sites but much needs to be done to reach standard readability levels. Finally, webmasters should play an active part in ensuring improved accuracy and readability of patient education materials.
